# Sixteenth Annual Course of Lectures in Rush Medical College

**Published:** 1858-10

**Authors:** 


### SIXTEENTH ANNUAL COURSE OF LECTURES IN RUSH MEDICAL COLLEGE.

The general introductory to the college session for 1858–9 was delivered by the professor of anatomy, Dr. J. W. Freer, on Monday evening, November 1st, to a larger class of students than has been present at the opening of the session for two or three years past. The lecture was well written, and in sentiment, strictly appropriate for the occasion; but as it will soon be published, we will not attempt to give our readers an outline of it at this time.

The term is now fairly commenced, each professor being at his post; and with two hospitals accessible to the students for regular clinical instruction, we think the facilities for acquiring a knowledge of medicine and surgery equal to that of any other school in our country.

